# Supplementary material for: Causal Relationship between Aging and Anorexia Nervosa: A White-Matter-Microstructure-Mediated Mendelian Randomization Analysis
Source: Biomedicines. 2024 Aug 16;12(8):1874. doi: 10.3390/biomedicines12081874 (PMC11351342; doi:10.3390/biomedicines12081874)
Supplement: Supplementary file 1 [file biomedicines-12-01874-s001.zip › Supplementary Figure/Figure S4/Singe SNP plot of MR analysis for TL to ubm-b-1542.pdf]

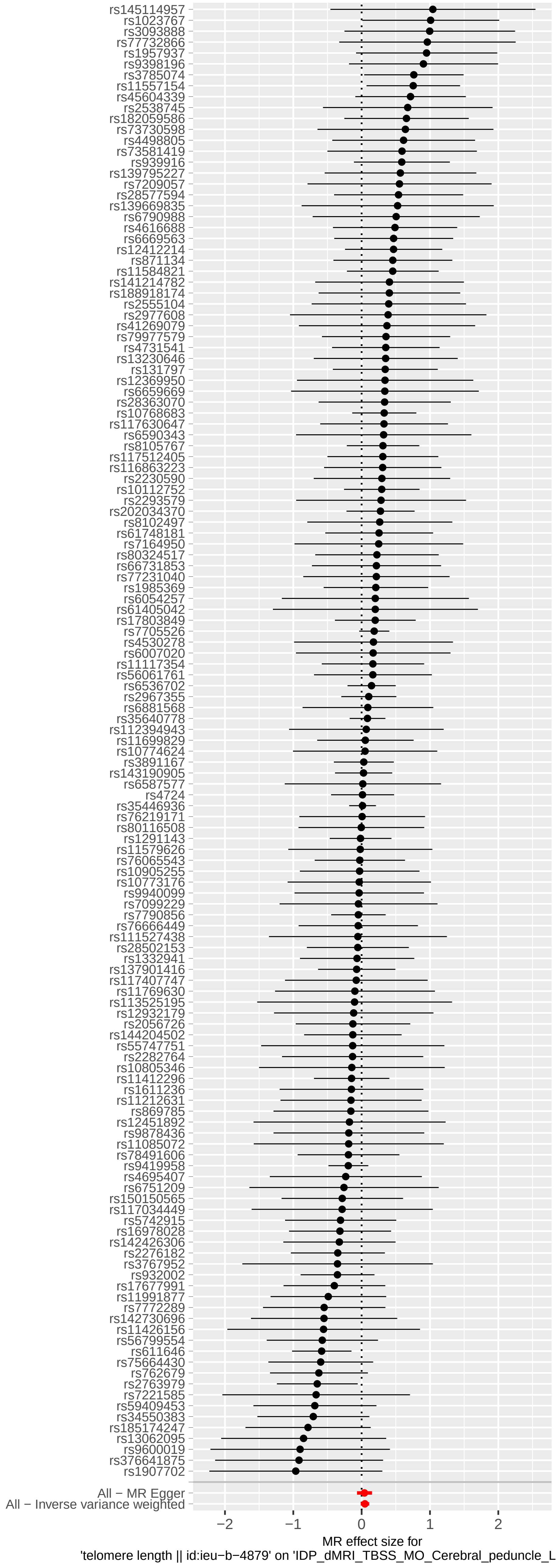

rs145114957
rs1023767
rs3093888
rs77732866
rs1957937
rs9398196
rs3785074
rs11557154
rs45604339
rs2538745
rs182059586
rs73730598
rs4498805
rs73581419
rs939916
rs139795227
rs7209057
rs28577594
rs139669835
rs6790988
rs4616688
rs6669563
rs12412214
rs871134
rs11584821
rs141214782
rs188918174
rs2555104
rs2977608
rs41269079
rs79977579
rs4731541
rs13230646
rs131797
rs12369950
rs6659669
rs28363070
rs10768683
rs117630647
rs6590343
rs8105767
rs117512405
rs116863223
rs2230590
rs10112752
rs2293579
rs202034370
rs8102497
rs61748181
rs7164950
rs80324517
rs66731853
rs77231040
rs1985369
rs6054257
rs61405042
rs17803849
rs7705526
rs4530278
rs6007020
rs11117354
rs56061761
rs6536702
rs2967355
rs6881568
rs35640778
rs112394943
rs11699829
rs10774624
rs3891167
rs143190905
rs6587577
rs4724
rs35446936
rs76219171
rs80116508
rs1291143
rs11579626
rs76065543
rs10905255
rs10773176
rs9940099
rs7099229
rs7790856
rs76666449
rs111527438
rs28502153
rs1332941
rs137901416
rs117407747
rs11769630
rs113525195
rs12932179
rs2056726
rs144204502
rs55747751
rs2282764
rs10805346
rs11412296
rs1611236
rs11212631
rs869785
rs12451892
rs9878436
rs11085072
rs78491606
rs9419958
rs4695407
rs6751209
rs150150565
rs117034449
rs5742915
rs16978028
rs142426306
rs2276182
rs3767952
rs932002
rs17677991
rs11991877
rs7772289
rs142730696
rs11426156
rs56799554
rs611646
rs75664430
rs762679
rs2763979
rs7221585
rs59409453
rs34550383
rs185174247
rs13062095
rs9600019
rs376641875
rs1907702
All − MR Egger
All − Inverse variance weighted
−2
−1
0
1
2
MR effect size for
'telomere length || id:ieu−b−4879' on 'IDP_dMRI_TBSS_MO_Cerebral_peduncle_L
